# Low-pressure endoscopy using the gel immersion method facilitates endoscopic reduction of a Morgagni hernia

**DOI:** 10.1055/a-2100-0506

**Published:** 2023-06-27

**Authors:** Keitaro Yano, Tomonori Yano, Alan Kawarai Lefor, Tetsuya Yano, Rei Aono, Hiroshi Sakaeda, Mitsuo Okada

**Affiliations:** 1 Department of Gastroenterology, Chikamori Hospital, Kochi, Japan; 2 Department of Medicine, Division of Gastroenterology, Jichi Medical University, Tochigi, Japan; 3 Department of Surgery, Jichi Medical University, Tochigi, Japan; 4 Kochi Clinic, Kochi, Japan

A Morgagni hernia is an uncommon diaphragmatic hernia. Complications such as obstruction and resulting necrosis can be life-threatening, and surgery is mandatory. However, minimally invasive treatments are preferred for elderly patients with comorbidities. Although treatment of a Morgagni hernia by endoscopic reduction has been reported 1 , hyperinflation during colonoscopy may increase intraluminal pressure and exacerbate incarceration. In the presence of an incarcerated Morgagni hernia, bowel preparation for colonoscopy is impossible, and securing the visual field is difficult. Gel immersion endoscopy has been reported to be useful for securing the visual field and decreasing intraluminal pressure 2 3 .

An 84-year-old woman with multiple cardiac comorbidities was hospitalized with epigastric pain and vomiting. A computed tomography scan showed prolapse of the transverse colon into the mediastinum and was consistent with obstruction due to a Morgagni hernia ( Fig. 1 ). Bearing in mind the patient’s comorbidities, surgical treatment was considered high risk. However, conservative treatment did not improve the obstruction. Colonoscopy without bowel preparation was performed ( Video 1 ). It was difficult to secure the visual field because of fecal impaction. However, by injecting gel (Viscoclear; Otsuka Pharmaceutical Factory, Tokushima, Japan), a transparent space between the tip of the endoscope and the intestinal wall was created and maintained, enabling endoscope insertion without gas insufflation while keeping the intraluminal pressure at a low level. A luminal constriction was found in the transverse colon, thought to be at the orifice of the Morgagni hernia ( Fig. 2 ). After insertion of the endoscope beyond the constriction, residual fluid and gas in the dilated lumen were aspirated ( Fig. 3 ). This reduced the herniation ( Fig. 4 ) and enabled endoscope insertion to the ileocecal valve, confirmed by X-ray fluoroscopy ( Fig. 5 ).

**Fig. 1:**
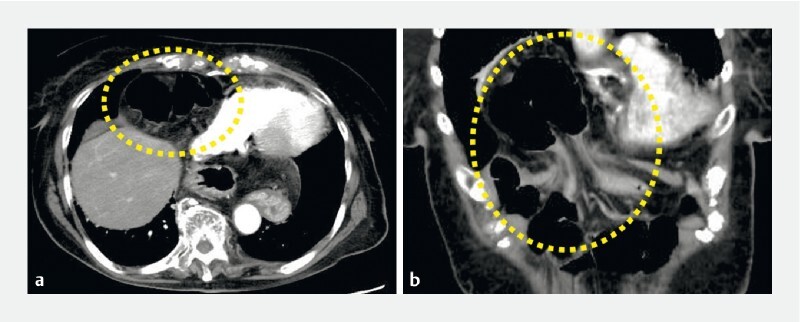
A computed tomography scan showed prolapse of the transverse colon into the mediastinum and was consistent with obstruction due to a Morgagni hernia. **a** axial view; **b** coronal view

**Video 1** A Morgagni hernia treated by means of endoscopic reduction with low-pressure endoscopy using the gel immersion method.


**Fig. 2:**
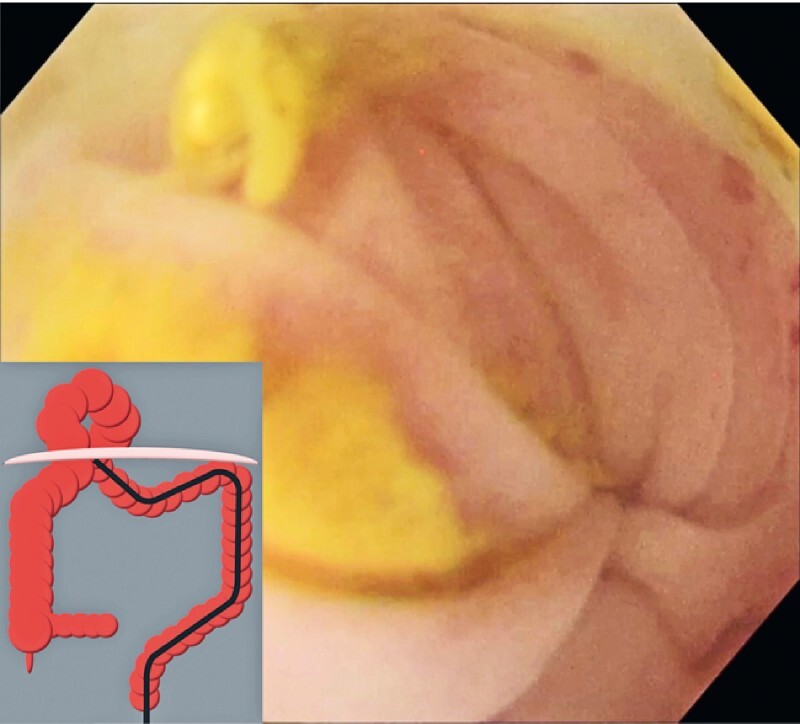
A luminal constriction was found in the transverse colon, which was thought to be at the orifice of the Morgagni hernia.

**Fig. 3:**
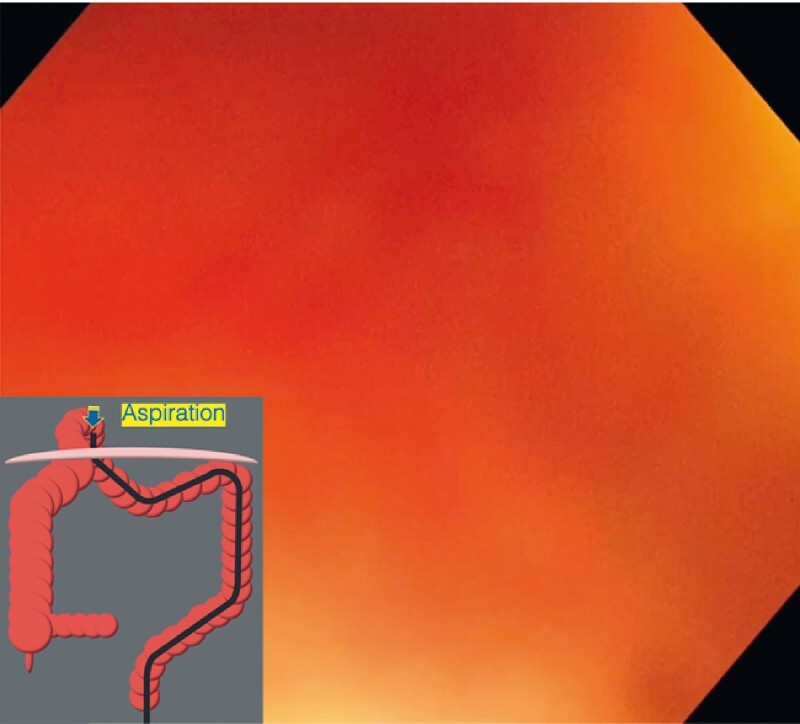
After insertion of the endoscope beyond the constriction, fecal retention was observed. Residual fluid and gas in the dilated lumen were aspirated, and the lumen collapsed.

**Fig. 4:**
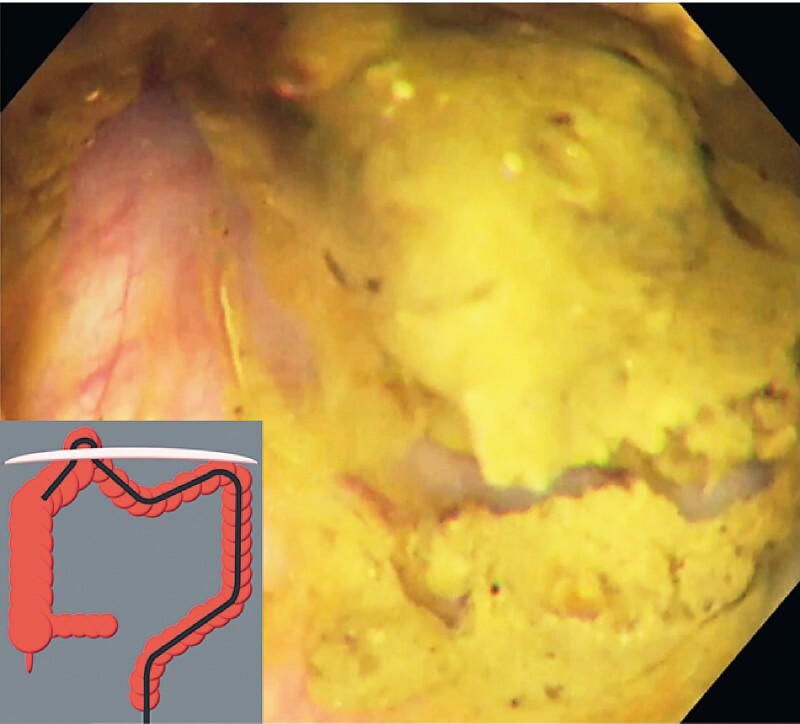
The collapsed lumen allowed endoscope insertion proximally, which reduced the herniation.

**Fig. 5:**
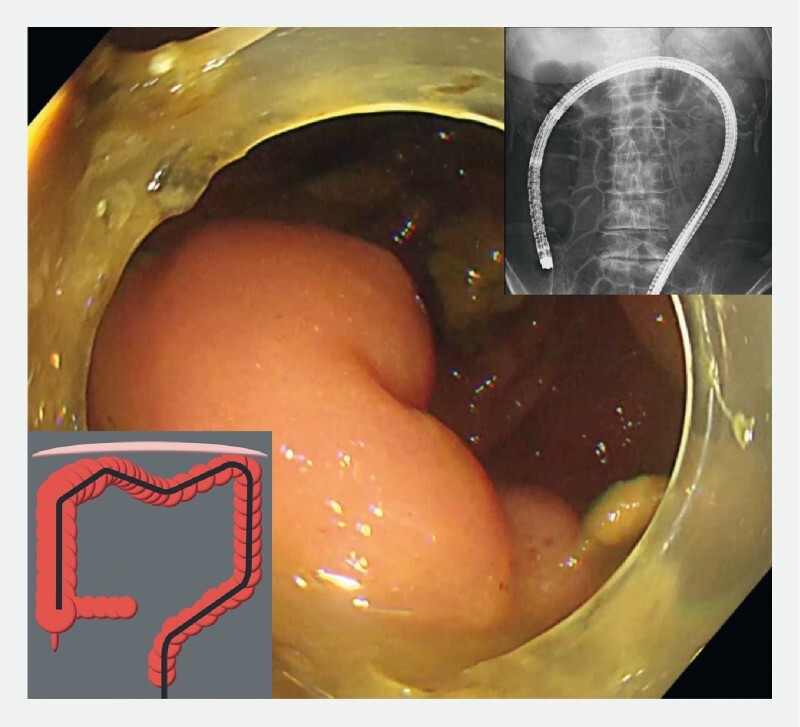
The endoscope was inserted to the ileocecal valve, and hernia reduction was confirmed by X-ray fluoroscopy.

Low-pressure endoscopy using the gel immersion method facilitates endoscopic reduction as a minimally invasive treatment of a Morgagni hernia.

Endoscopy_UCTN_Code_CCL_1AD_2AJ

## Notes

Endoscopy E-Videos

https://eref.thieme.de/e-videos
*E-Videos* is an open access online section of the journal *Endoscopy* , reporting on interesting cases and new techniques in gastroenterological endoscopy. All papers include a high-quality video and are published with a Creative Commons CC-BY license. Endoscopy E-Videos qualify for HINARI discounts and waivers and eligibility is automatically checked during the submission process. We grant 100% waivers to articles whose corresponding authors are based in Group A countries and 50% waivers to those who are based in Group B countries as classified by Research4Life (see: https://www.research4life.org/access/eligibility/ ). This section has its own submission website at https://mc.manuscriptcentral.com/e-videos
